# Pituitary Diseases and COVID-19 Outcomes in South Korea: A Nationwide Cohort Study

**DOI:** 10.3390/jcm12144799

**Published:** 2023-07-20

**Authors:** Jeonghoon Ha, Kyoung Min Kim, Dong-Jun Lim, Keeho Song, Gi Hyeon Seo

**Affiliations:** 1 Division of Endocrinology and Metabolism, Department of Internal Medicine, Seoul St. Mary’s Hospital, College of Medicine, The Catholic University of Korea, Seoul 06591, Republic of Korea; 3002041@catholic.ac.kr (J.H.);; 2 Division of Endocrinology, Department of Internal Medicine, Yongin Severance Hospital, Yonsei University College of Medicine, Yongin 16995, Republic of Korea; 3 Division of Endocrinology and Metabolism, Department of Internal Medicine, Konkuk University School of Medicine, Seoul 05030, Republic of Korea; 4 Health Insurance Review and Assessment Service, Wonju 26465, Republic of Korea

**Keywords:** COVID-19, pituitary disease, mortality

## Abstract

The pituitary gland is either directly or indirectly impacted by SARS-CoV-2 infection. As a consequence of SARS-CoV-2 infection, hypothalamic–pituitary dysfunction or pituitary apoplexy can occur. This study aimed to investigate severe COVID-19 outcomes and COVID-19-related mortality in patients with underlying pituitary disease in Korea using a nationwide cohort database. The data required for this study were obtained from the Health Insurance Review and Assessment Service of Korea. Patients with SARS-CoV-2 infection between January 2020 and December 2021 were divided into the following three groups and analyzed: Group A, those who were hospitalized for SARS-CoV-2 infection without underlying pituitary disease (n = 725,170); Group B, those who were hospitalized for SARS-CoV-2 infection with underlying pituitary disease without exposure to systemic steroids (n = 1509); and Group C, patients with underlying pituitary disease and exposure to systemic steroids (n = 365). Differences in severe COVID-19, requirement for oxygen therapy, intensive care unit admission, application of invasive ventilation or use of extracorporeal membrane oxygenation, and COVID-19-related deaths between groups were then analyzed. Group C had the highest rates of hospitalization after COVID-19 infection (82.2%) and mortality within 30 days of infection (6.8%). Group B had a 1.3-fold increase in severe COVID-19 outcomes compared to Group A. Group C had 1.8-fold and 1.3-fold increases in severe COVID-19 outcomes compared to Group A and Group B, respectively. Group C also had 2.34 and 3.24 times higher mortality rates within 30 days of COVID-19 infection than Group A and Group B, respectively. In conclusion, patients with pituitary disease who are receiving systemic steroids have poorer outcomes and higher mortality associated with COVID-19. Therefore, thorough COVID-19 infection control is required in these patients.

## 1. Introduction

Since the first COVID-19 case was reported in November 2019, the disease rapidly evolved into a global pandemic that was declared by the World Health Organization in March 2020. South Korea reported its first case of COVID-19 in January 2020. Since then, there have been a total of 32,256,154 confirmed cases and 35,071 deaths as of 4 July 2023.

Although COVID-19 primarily affects the respiratory system, it is also linked to a wide range of extrapulmonary manifestations, including vascular, cardiac, hepatic, gastrointestinal, and dermatological complications [1,2]. Recent evidence suggests that the pituitary gland might also be directly or indirectly impacted by SARS-CoV-2 infection [3], potentially leading to hypothalamic–pituitary dysfunction or pituitary apoplexy [3,4,5,6]. The implications of angiotensin-converting enzyme 2, a receptor for SARS-CoV-2 that is ubiquitously expressed on various cellular surfaces, including those of the pituitary, on pituitary dysfunction post-COVID-19 infection have been reported [7]. Dysnatremia, particularly hyponatremia, has been identified as a possible marker of severe COVID-19 infection [8]. Patients with pituitary disease often present with obesity and diabetes, both of which have been associated with higher mortality rates and worse outcomes in COVID-19 cases [9]. However, establishing a causal relationship between COVID-19 and poor outcomes in patients with pituitary disease is challenging due to the rarity of the condition [10]. While it can be hypothesized that COVID-19 might be linked to a poor prognosis in patients with pituitary disease, no studies have yet confirmed this association, especially in the Korean population. Determining COVID-19 outcomes in this population is crucial for guiding future patient care and healthcare policies, particularly because systemic steroid use is prevalent among patients with pituitary disease as a result of adrenocorticotropic hormone deficiency. To address this knowledge gap, the present study aimed to examine the outcomes and severity of COVID-19-related mortality in Korean patients with pituitary disorders, with a particular focus on those who received systemic steroids. We utilized a nationwide cohort database to provide a comprehensive analysis. The results of this study will contribute to our understanding of how pituitary diseases might influence the clinical course of COVID-19 infection.

## 2. Materials and Methods

### 2.1. Data Source

The data used for this study were obtained from the Health Insurance Review and Assessment Service, which was established to develop an accurate claim review and quality assessment system for the National Health Insurance (NHI) in Korea. The NHI is the only public health insurance in Korea. It is operated by the Ministry of Health and Welfare. All clinics and hospitals in Korea must submit patient data, including diagnostic and medical cost information, to the National Health Insurance Service for patient care claims. Informed consent was waived for this study, as it utilized publicly available, anonymized, and deidentified data. Analyses of SARS-CoV-2 infections conducted in the same manner as this study have already been reported [11,12].

### 2.2. Study Population

In this retrospective cohort study, a total of 727,044 patients hospitalized for SARS-CoV-2 infection from January 2020 to December 2021 were analyzed. These patients were divided into three groups: Group A, patients who were hospitalized for SARS-CoV-2 infection without underlying pituitary disease; Group B, patients who were hospitalized for SARS-CoV-2 infection with underlying pituitary disease without systemic steroid exposure; and Group C, patients with underlying pituitary disease and exposure to systemic steroids. SARS-CoV-2 infection was confirmed according to the WHO guideline using a positive test result from a real-time RT-PCR assay of nasal or pharyngeal swabs [13]. Patients with COVID-19 were identified using the following ICD-10 codes: B34.2 (coronavirus infection, unspecified site), U18.1 (novel coronavirus infection), U07.1 (COVID-19, virus identified), and U07.2 (COVID-19, virus not identified). Patients with underlying pituitary diseases were identified based on the International Classification of Diseases, Tenth Revision (ICD-10) codes, having at least one claim within one year after the index date. The following specific ICD-10 codes were used to define each pituitary disease: D35.2 for nonfunctioning pituitary adenoma, E22.0 and D35.2 for acromegaly, E22.1 and D35.2 for prolactinoma, E24 and D35.2 for Cushing’s disease, and E23.0 with/without D35.2 for hypopituitarism. Hypopituitarism was coded when there was a deficiency of three or more anterior pituitary hormones. Cases associated with or without a pituitary mass, such as Sheehan’s syndrome and empty sella syndrome, were included in the analysis. Cushing’s syndrome (E24.2, E24.8, E24.9) and hyperprolactinemia (E221) were excluded from the analysis. The use of these ICD-10 codes was based on a recently proposed large data research methodology using the Korean National Health Information Database [14]. Comorbidities, including hypertension, diabetes, COPD or asthma, and liver cirrhosis, were defined as having at least one claim in either outpatient, inpatient, or both settings using appropriate ICD-10 codes within one year prior to COVID-19 confirmation. Systemic steroid administration was defined as the administration of oral prednisolone at a dose of ≥5 mg/day or the equivalent for at least 30 days within 180 days preceding COVID-19 diagnosis [15].

### 2.3. Outcomes

The aim of this study was to determine the following severe COVID-19 outcomes among patients with underlying pituitary disease, both with and without systemic steroid exposure, in comparison with patients without underlying pituitary disease: hospitalization rate, need for intensive care unit (ICU) admission, requirement for oxygen therapy (including mechanical ventilation or the use of extracorporeal membrane oxygenation), and death within 30 days after COVID-19 infection.

### 2.4. Statistical Analysis

Baseline characteristics and clinical outcomes of the study population are presented as numbers with percentages (%) and means with standard deviations (SD). Continuous variables were compared between two groups using the t-test, while categorical variables were analyzed using the chi-square test or Fisher’s exact test, as appropriate. Logistic regression analysis was used to calculate odds ratios (OR) and 95% confidence intervals (CI) for mortality in patients with pituitary diseases with or without systemic steroids. A multivariable-adjusted OR was obtained from logistic regression analysis to compare mortality rates between groups, accounting for age, sex, and comorbidities. Statistical significance was set at a two-sided *p*-value of less than 0.05. All statistical analyses were performed using R version 4.1.1 (The R Project for Statistical Computing, Vienna, Austria).

## 3. Results

A total of 727,044 COVID-19 cases during the study period were analyzed. Group C had the highest mean age, at 61.3 ± 16.8 years. It also had the highest prevalence of comorbidities, such as COPD or asthma (15.9%), liver cirrhosis (3.0%), diabetes (11.2%), and hypertension (50.4%), among the three groups (Table 1).

Among the pituitary diseases, nonfunctioning pituitary adenoma was the most prevalent in both Group B (51.2%) and Group C (37.2%), with prolactinoma being the second most common pituitary disorder in Group B (43.7%) (Table 1). The proportion of patients hospitalized in a general ward after contracting COVID-19 was the lowest in Group A (63.2%) but the highest in Group C (82.2%) (Table 2). Moreover, Group C had the highest percentage of ICU admissions (13.7%) as well as the highest percentage of patients requiring oxygen therapy (34.2%) or extracorporeal membrane oxygenation (ECMO) (8.2%) among the three groups (Table 2).

The risk of severe COVID-19 outcomes was 1.31 times higher (95% CI: 1.14–1.52) for Group B compared to Group A and 1.8 times higher (CI: 1.43–2.27) for Group C compared to Group A in models adjusted for age, sex, and comorbidities (Table 3). When comparing severe COVID-19 outcomes with and without steroids among patients with pituitary disease, we observed a 1.64 times higher risk (95% CI: 1.25–2.17) in Group C compared to Group B (Table 3). The 30-day mortality rate after COVID-19 infection was higher in Group C (6.8%) than in Group A (1.2%) and Group B (1.2%) (Table 2). In the adjusted model, there was a 2.34 times higher risk (95% CI: 1.53–3.57) of mortality within 30 days of COVID-19 infection in Group C compared to Group A and a 3.24 times higher risk (95% CI: 1.70–6.18) compared to Group B (Table 3).

## 4. Discussion

**Figure 1:**
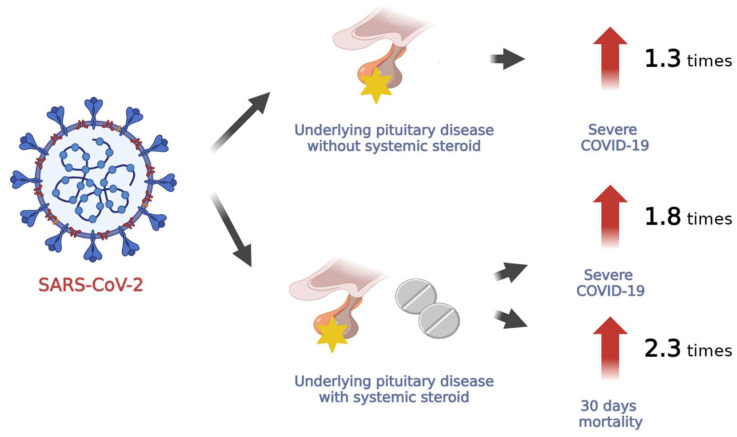
Impact of COVID-19 infection on the prognosis of patients with pituitary disease.

In this study analyzing 727,044 individuals, it was discovered that those with pituitary disease had a higher likelihood of experiencing severe morbidity due to COVID-19 infection. Moreover, there was an increased risk of severe COVID-19 outcomes and mortality associated with COVID-19 for those exposed to systemic steroids (Figure 1).

Since the beginning of the pandemic, there have been reports addressing the impact of COVID-19 infection on this population. Reports have indicated that COVID-19 infection might lead to pituitary apoplexy, hyponatremia, or hypophysitis [9]. It has been proposed that pituitary disease might be linked to severe COVID-19 infection, as it is frequently accompanied by diabetes, obesity, and vertebral fractures known to be associated with severe COVID-19 infection [16]. In the realm of pituitary disorders, several cases of pituitary apoplexy have been documented following COVID-19 infection [2]. Solori-Pineda and colleagues reported the case of a 27-year-old female patient who developed a frontal headache and dizziness post-COVID-19 infection. Pituitary imaging revealed a pituitary macroadenoma. Unfortunately, the patient’s condition deteriorated, leading to a decline in respiratory function and eventually resulting in her death [5]. However, there is currently no large-scale, real-world evidence demonstrating a connection between COVID-19 infection and increased severity or mortality in patients with pituitary disease.

In our study, we found that COVID-19 infection led to increased hospitalization and ICU admission rates for patients with pituitary disease on systemic steroids compared to those without pituitary disease or those with pituitary disease without taking systemic steroids. Furthermore, there were increased rates of severe COVID-19, characterized by a higher need for oxygen therapy, invasive ventilation, or ECMO. Surprisingly, the 30-day mortality rate was 2.34 times higher in patients with pituitary disease and exposure to systemic steroids than in those with COVID-19 without pituitary disease and 3.24 times higher than in those with pituitary disease but not exposed to systemic steroids. Naturally, patients with pituitary disease receiving systemic steroids were older and more likely to have comorbidities, such as respiratory diseases like COPD and asthma, liver cirrhosis, diabetes, and hypertension, compared to the other groups. However, even after adjusting for these factors, the poor prognosis for COVID-19 infection remained unchanged.

The significant increase in severe COVID-19 outcomes and mortality among pituitary patients with exposure to systemic steroids compared to those without such exposure might be attributed to the following reasons: The fundamental role of glucocorticoids in impairing the immune system heightens vulnerability to infections [17,18]. Furthermore, patients with adrenal insufficiency exhibit a higher propensity for encountering infection-induced adrenal crises than those without such insufficiency, which constitutes a grave complication that might result in fatality [19,20,21]. Some reports have also suggested that long-term steroid use can impair viral clearance, leading to persistent COVID-19 viral replication while decreasing interferon production, which in turn can increase the risk of bacterial and other secondary infections [22,23]. Consequently, several studies have already established a link between long-term systemic steroid administration and an unfavorable prognosis for COVID-19 infection [24,25,26]. Ku et al. have recently shown that long-term exposure to systemic steroids can increase the mortality and severity of COVID-19 in a Korean nationwide cohort database of COVID-19 patients [11]. Ward et al. have demonstrated that exposure to glucocorticoids (cumulative prednisolone-equivalent dose <2000 or ≥2000 mg) within 120 days is linked to heightened risks of hospitalization and mortality after COVID-19 infection [27].

Hypocortisolism might have also played a role in mortality, as steroid-treated patients might have failed to adjust their steroids appropriately based on the sick days rule. Multiple guidelines recommend that individuals with adrenal insufficiency should employ the sick days rule and increase the existing dose of glucocorticoids during COVID-19 infection [16,21,28,29]. Moreover, some studies have indicated that administering high doses of steroids is effective in augmenting the duration of ventilation-free days and mitigating mortality [30,31]. It is important to note that these studies did not exclude individuals with pituitary disorders, nor did they explore specific factors related to severe COVID-19 outcomes. Additional studies are necessary to establish whether adverse outcomes are due to the negative impact of steroid administration itself or due to an inability to appropriately adjust steroid doses in the context of an infection.

Considering that patients with severe COVID-19 tend to have pre-existing cardiovascular and metabolic comorbidities, such as diabetes, hypertension, and obesity [32,33,34], it is crucial to take these factors into account when evaluating the prognosis of individuals with pituitary diseases who contract COVID-19. The most frequent phenotype of COVID-19 patients requiring hospitalization is older patients (60–70 years) with metabolic comorbidities, such as hypertension (30–55%) and diabetes (15–33%), which is consistent with our study findings [16]. Previous reports have indicated that diabetes is the common comorbidity among hospitalized patients with COVID-19 infection and that poor glycemic control has a negative impact on disease outcomes [35,36]. Patients with COPD are at an elevated risk of developing severe pneumonia and experiencing unfavorable outcomes when infected with COVID-19, potentially due to diminished lung reserves or heightened expression of ACE-2 receptors in the small airways [37]. COVID-19 infection can lead to an evident proinflammatory cytokine profile, which is linked to an unfavorable prognosis in individuals with cirrhosis [38]. COVID-19 infection has been shown to activate the renin–angiotensin system. Hypertension is also linked to an unfavorable prognosis for COVID-19 [39]. Hence, in our study, it is challenging to attribute a single cause to an unfavorable prognosis of COVID-19 infection in patients with pituitary disease and exposure to systemic steroids.

While this study primarily examined adrenocorticotropic hormone deficiency within the context of anterior pituitary hormones, other hormone deficiencies, such as thyroid-stimulating hormone, might also exist. However, adrenocorticotropic hormone deficiency remains the predominant contributor to mortality in patients with nonfunctioning pituitary adenomas and in individuals with acromegaly, among the anterior pituitary hormones [40,41]. We have also observed changes in the autonomic nervous system associated with hormone deficiency in patients with nonfunctioning pituitary adenomas using heart rate variability as an assessment tool. Our findings revealed that simultaneous adrenocorticotropic hormone deficiency could lead to increased autonomic dysfunction [42]. Therefore, steroid supplementation in patients with pituitary disease indicates adrenocorticotropic hormone deficiency, which could potentially serve as a marker of increased mortality risk.

This study has several limitations that warrant cautious interpretation. First, the diagnosis of pituitary disease was based on claims data using the ICD-10 code. In Korea, the National Health Insurance Service categorizes pituitary disease as a rare disorder that carries a substantial medical cost burden. It has implemented a system to reduce treatment expenses. Consequently, diagnosis codes for pituitary diseases are managed with precision. Moreover, the pituitary disease-related codes employed in this study have been officially recommended by the Korean Endocrine Society for utilization in large-scale research on endocrine diseases within Korea [14]. Second, the retrospective analysis design makes it challenging to establish a direct relationship between COVID-19 infection and the severity or mortality of the disease. Third, as this study utilized claim data, there was a lack of information on medical records and laboratory test results, thus limiting the definition of severe COVID-19 to the need for oxygen therapy, invasive mechanical ventilation, or admission to the ICU. Fourth, we were unable to analyze pituitary diseases by subtype. Due to the small number of patients with certain pituitary disorders, we focused on individuals with pituitary diseases as a whole rather than examining them as separate conditions. In the future, the impact of COVID-19 infection on individual diseases needs to be assessed. Building on previously noted limitations, determining the disease control status using the current data structure proves challenging. For example, in acromegaly, the response to COVID-19 infection might differ based on the disease control status. However, the existing data structure does not permit the identification of all such instances. Fifth, quantitative analysis of SARS-CoV-2 infection was not feasible. Outcomes may vary depending on viral load, necessitating further follow-up studies to explore this aspect. Lastly, it does not account for comorbidities that could potentially impact the outcome of a COVID-19 infection, like cancer or HIV. However, we have made concerted efforts to include significant comorbidities, such as diabetes, CKD, COPD, and liver cirrhosis. Furthermore, we made an effort to correct for age in order to minimize its influence on our findings.

## 5. Conclusions

To the best of our knowledge, this study presents the first large-scale analysis in South Korea examining the risk of severe COVID-19 outcomes in patients with underlying pituitary diseases. The risk of severe COVID-19 outcomes and 30-day mortality is heightened in patients with pituitary diseases, particularly those who are on systemic steroids. Our findings highlight the significance of acknowledging the poor prognosis of COVID-19 in pituitary patients, regardless of etiologies, proactively preventing COVID-19 infection in this specific group, and closely monitoring their clinical status if infected. Further research is needed to determine how COVID-19 outcomes vary based on the cause of pituitary disease and the level of disease control.

## Figures and Tables

**Table 1:** Baseline characteristics of the study population.

	Group A (n = 725,170)	Group B (n = 1509)	Group C (n = 365)
Mean age (years)	48.2 ± 17.5	47.0 ± 17.1	61.3 ± 16.8
<60 years, %	70.9%	74.9%	36.7%
≥60 years, %	29.1%	25.1%	63.3%
Female	50.0%	75.2%	62.2%
Comorbidity, n (%)			
COPD or asthma	38,266 (5.3)	117 (7.8)	58 (15.9)
Liver cirrhosis	3972 (0.5)	13 (0.9)	11 (3.0)
Diabetes	11,841 (1.6)	57 (3.8)	41 (11.2)
CKD	9427 (1.3)	54 (3.6)	27 (7.5)
Hypertension	116,111 (16.0)	396 (26.3)	184 (50.4)
Underlying pituitary disease, n (%)			
Nonfunctioning pituitary adenoma	0 (0)	773 (51.2)	136 (37.2)
Acromegaly	0 (0)	28 (1.9)	15 (4.1)
Prolactinoma	0 (0)	659 (43.7)	15 (4.1)
Cushing’s disease	0 (0)	7 (0.5)	8 (2.2)
Hypopituitarism	0 (0)	239 (15.8)	194 (53.2)

Data are n (%); Group A, SARS-CoV-2 (+) without any underlying pituitary disease; Group B, SARS-CoV-2 (+) with underlying pituitary disease but no systemic steroid exposure; Group C, SARS-CoV-2 (+) with underlying pituitary disease and exposure to systemic steroids. Percentages might not sum to 100% due to the co-existence of underlying pituitary diseases. COPD, chronic obstructive pulmonary disease; CKD, chronic kidney disease (stage 3b–5).

**Table 2:** Severe COVID-19 outcomes or COVID-19-related death.

	Group A (n = 725,170)	Group B (n = 1509)	Group C (n = 365)
Severe SARS-CoV-2			
General ward admission	63.2%	67.9%	82.2%
ICU admission	3.1%	4.3%	13.7%
Requirement for oxygen therapy	13.1%	16.7%	34.2%
Requirement for mechanical ventilation or ECMO	1.4%	2.3%	8.2%
Any of the above	13.4%	17.2%	35.3%
Death within 30 days after infection	1.2%	1.2%	6.8%

Data are %; Group A, SARS-CoV-2 (+) without any underlying pituitary disease; Group B, SARS-CoV-2 (+) with underlying pituitary disease but no systemic steroid exposure; Group C, SARS-CoV-2 (+) with underlying pituitary disease and exposure to systemic steroids. ECMO, extracorporeal membrane oxygenation.

**Table 3:** Odds ratios of severe COVID-19 outcomes and COVID-19-related death.

	Group A	Group B	Group C
Severe COVID-19			
Odds ratio	Ref.	1.34 (1.17–1.53)	3.54 (2.85–4.39)
		Ref.	2.64 (2.05–3.40)
Adjusted * odds ratio	Ref.	1.31 (1.14–1.52)	1.80 (1.43–2.27)
		Ref.	1.64 (1.25–2.17)
Death within 30 days after infection			
Odds ratio	Ref.	1.00 (0.63–1.59)	6.07 (4.04–9.12)
		Ref.	6.07 (3.27–11.25)
Adjusted * odds ratio	Ref.	0.89 (0.55–1.43)	2.34 (1.53–3.57)
		Ref.	3.24 (1.70–6.18)

* adjusted by age group, sex, and comorbidities.

## Data Availability

The datasets generated and/or analyzed during the current study are not publicly available due to personal data protection legislation but are available from the first author or corresponding author on reasonable request.
